# A Longitudinal Qualitative Study of Resourcefulness Training Needs During the Hospital‐to‐Home Transition After Stroke in Young and Middle‐Aged Adults

**DOI:** 10.1111/hex.70462

**Published:** 2025-10-29

**Authors:** Juan Sun, Yali Li, Yongmei Wang, Yilin Du, Hui Shen, Sijia Zhang, Hongru Wang, Huimin Zhang

**Affiliations:** ^1^ Xinxiang Medical University Xinxiang People's Republic of China; ^2^ Xinxiang First People's Hospital Xinxiang People's Republic of China

**Keywords:** hospital‐to‐home transition, longitudinal qualitative study, resourcefulness training, transitional care, young and middle‐aged stroke survivors

## Abstract

**Introduction:**

Resourcefulness training (RT) supports stroke recovery by improving coping strategies and self‐management. Recognising the need for such training is crucial for providing targeted transitional care. We aimed to explore the evolving need for RT among young and middle‐aged stroke survivors during their transition from hospital to home.

**Methods:**

Using purposive sampling, we conducted two rounds of semi‐structured face‐to‐face interviews, supplemented by observational methods, involving 21 young and middle‐aged stroke survivors and 13 informal caregivers. Data were collected at two time points: 1–3 days before hospital discharge (T1) and 2 months after returning home (T2). Thematic analysis was conducted, and NVivo 12 was used to facilitate data coding and organisation.

**Results:**

Longitudinal qualitative analysis revealed two interconnected and fluid psychosocial stages in the transition from hospital to home: a vulnerable window immediately after discharge and an open period for rebuilding the rhythm of life. Each stage encompassed two major themes and four sub‐themes: (1) crisis awareness: limited coping capacity and concerns regarding stroke recovery; (2) low participation in rehabilitation training: limited initiative towards rehabilitation and insufficient awareness of available social resources; (3) repositioning needs: adjustment and reconstruction of self‐perception, along with a desire for self‐actualisation; and (4) desire for social support: assistance with behavioural change and the pursuit of community acceptance.

**Conclusion:**

These findings indicate that the need for RT for young and middle‐aged stroke survivors during the hospital‐to‐home transition evolves dynamically from personal to social resourcefulness. Enhancing disease awareness during hospitalisation, gradually integrating social support resources after discharge, and tailoring interventions according to age, sex and rehabilitation status may better meet the rehabilitation needs of this population as they transition to home care.

**Patient or Public Contribution:**

Patients and caregivers were actively involved in the feedback and early phases of data analysis. They verified the accuracy and relevance of the analysed themes. The medical staff provided respondent names and bed information that met the inclusion criteria. Their involvement contributed to ensuring the accuracy of data collection and enhancing the validity of the results.

## 1 Introduction

Stroke is a major global public health challenge, with approximately 70%–80% of survivors living with varying degrees of functional impairment [1]. High rates of disability and recurrence continue to place a substantial burden on patients, families and healthcare systems. A recent study across 38 countries in the Americas indicated that the total number of stroke cases is increasing annually and shows a clear trend towards younger patients [2]. In China, the world's most populous country, the lifetime risk of stroke is 39.3%, the highest globally, with individuals aged 18–64 years accounting for 28.7% of cases [3]. This indicates that stroke is no longer confined to its traditional classification as a disease of older adults but has emerged as a major threat to the health and working capacity of young and middle‐aged adults. Following acute treatment, most stroke survivors engage in home‐based rehabilitation [4]. The first 1–3 months after discharge, the hospital‐to‐home transition period, is critical in determining the success or failure of rehabilitation [5]. During this 90‐day window, patients must simultaneously rebuild their self‐care abilities to address post‐stroke functional impairments, prevent complications, reduce the risk of readmission, and reshape their social identities to reintegrate into community life [6]. Resourcefulness training (RT), an emerging rehabilitation intervention grounded in cognitive–behavioural dual pathways, has the potential to enhance stroke survivors' self‐management abilities and coping strategies, strengthen psychological resilience, and facilitate the shift from passivity to proactivity during the hospital‐to‐home transition [7].

RT is an intervention strategy derived from the theory of resourcefulness and aims to strengthen patients' capacity for adaptation and coping [8]. It is grounded in two complementary concepts proposed by Zauszniewski (2006): personal resourcefulness, defined as the ability to perform daily tasks independently, and social resourcefulness, defined as the ability to seek help from others when tasks cannot be managed alone [9]. Both dimensions are regarded as essential under adverse conditions. Building on this framework, several countries have established integrated curriculum–tools–evidence‐based systems that incorporate RT into transitional care management for chronic diseases, including cancer, cardiovascular disease and cerebrovascular disease [10, 11]. A representative model is the Care Transition Intervention, which simultaneously empowers patients and their families to achieve continuous improvement in self‐management [12]. Although RT was introduced relatively late in China, its application has expanded from cancer and diabetes to include stroke. Preliminary findings suggest that it can enhance self‐management, reduce anxiety and depression, and improve quality of life [13, 14]. However, existing research has provided limited insight into the dynamic development of resourcefulness and RT. Most interventions remain cross‐sectional in design, resulting in homogenised training programmes. Connor et al. [15] synthesised 14 qualitative studies on early discharge after stroke, highlighting significant gaps in support and transitional services. However, the absence of longitudinal follow‐up makes it challenging to accurately capture patients' rehabilitation trajectories and evolving needs.

Longitudinal qualitative research involves conducting multiple interviews with the same participants over time to record and analyse trends, mechanisms and individual differences in patient needs, thereby supporting the design of precise intervention plans [16]. Two longitudinal qualitative studies by Chen et al. [17] and Lin et al. [6] revealed that stroke rehabilitation is not a smooth upward trajectory, but rather a life journey characterised by disruption, reshaping and adaptation. Building on this perspective, the present study applied longitudinal qualitative research to the stroke transition period to capture the evolving needs related to psychological resilience and functional recovery. This approach is essential for developing targeted and adaptive RT, achieving a closed loop of stage–needs–training, and ultimately enhancing both resourcefulness and rehabilitation outcomes in stroke survivors.

We aimed to explore the dynamic need for RT among young and middle‐aged stroke survivors during their transition from hospital to home.

## 2 Materials and Methods

### 2.1 Study Design and Theoretical Framework

The study adhered to ethical principles and was approved by the Ethics Committee of Xinxiang Medical University (Approval No.: [XYLL‐20240352]) (Appendix 1). All participants provided written informed consent before participation, ensuring that they fully understood the study′s purpose, procedures and rights. Participant privacy was protected, and all data were treated confidentially. We conducted a prospective, longitudinal qualitative study grounded in Husserl's descriptive phenomenology as the methodological foundation [18, 19]. Phenomenology reframes resourcefulness as an abstract indicator of the ability to engage in a dynamic psychological, social and temporal practice of survival. By bracketing preconceived categories, it seeks to capture patients' lived experiences of resourcefulness needs during the transition period and their evolving trajectory [20]. Develop interview guidelines based on resourcefulness theory and adhere to the interview standards for PRO scales proposed by Brédart et al. [21]. The interview timing was structured to capture meaningful changes in participants' experiences [22]. Semi‐structured, face‐to‐face interviews were conducted with stroke survivors and their caregivers upon discharge (T1) and about 2 months post‐discharge (T2), supplemented by participatory observation. The study was conducted and reported in accordance with the Consolidated Criteria for Reporting Qualitative Research (COREQ), developed by the School of Public Health, University of Sydney, in 2007 [23].

### 2.2 Participants and Recruitment

The design phase of this study focused on the subjective experiences of patients and their families and therefore did not include formal caregivers in the sampling framework. The participants were young and middle‐aged individuals who had experienced a stroke, along with their family caregivers, recruited from a tertiary hospital between October and December 2024. The hospital admits thousands of patients with stroke annually and serves a population with diverse cultural, linguistic and socio‐economic backgrounds. The demographic and clinical characteristics of the sample were comparable to those of the concurrently hospitalised stroke population. Purposive sampling was used to recruit participants from three neurology wards and two rehabilitation wards. The inclusion and exclusion criteria for both patients and caregivers are presented in Table 1.

**Table 1:** Study inclusion and exclusion criteria for participants.

	Inclusion criteria	Exclusion criteria
Patient	Hospitalised patients aged 18–64 with a first stroke.	With severe organic lesions.
	Transitioning from hospital to home.	Living in a residential aged care facility.
	Barthel Index > 40, normal cognitive function, able to communicate verbally.	Missing patients.
Caregiver	Unpaid informal caregiver to a patient transitioning from hospital to home.	Caregivers of a patient living in a residential aged care facility.
	Aged 18 years and older.	Missing caregiver.
	In good health.	

### 2.3 Data Collection

Table 2 presents the interview outline developed by a panel of four senior experts (M.D.). Guided by the dual structure of personal and social resourcefulness in resourcefulness theory, the outline directly informed the dimensions of the interview questions [24]. Questions 1–6 explored patients' ability to manage their illness independently, whereas questions 7–8 focused on their behaviour in seeking external support. Together, these questions captured patients' needs for self‐management at the individual level and support at the social level. To refine this outline, three pilot interviews were conducted with young and middle‐aged stroke survivors (including one caregiver) who met the inclusion criteria. Based on these interviews, the order and timing of the questions were adjusted, and techniques for recording field notes were optimised. The patients and caregivers were interviewed separately using the same outline. Patients responded in the first person, describing their own experiences (e.g., ‘I don′t know much about strokes′), whereas caregivers provided accounts of the patient's experiences using the third person (e.g., ‘He had very little knowledge about strokes′). The same interview outline was applied at T1 and T2, with minor adjustments at T2 to reflect the participants' evolving circumstances.

**Table 2:** Outline of the interview.

Theoretical framework	Thematic	Synopsis of the interview
Personal Resourcefulness	Remedial self‐control	1. What do you currently know about stroke disease? How much?
		2. How has a stroke affected your life? How have you managed to cope with these effects?
	Modified problem‐solving skills	3. What do you do when faced with a disease problem you can't solve using your usual thinking and approach?
		4. What behaviours or habits do you believe are currently contributing to your illness? What actions will you take to improve?
	Perceived self‐efficacy	5. Describe the emotional changes you have experienced since your illness and how you see yourself.
		6. How do you self‐regulate in the face of negative emotions?
Social Resourcefulness	Finding social support	7. Do you reach out for help when you are sick? What kind of help do you usually seek from family, friends, healthcare workers or social organisations?
		8. Do you take the initiative to participate in some community health promotion and public lectures when you return to the community after recovery? How active do you think you are?

*Note:* T1: 1–3 days before discharge; T2: 7–9 weeks after discharge.

Two interviewers independently conducted one‐on‐one, face‐to‐face, open‐ended interviews. Additionally, they participated in clinical rounds and daily nursing care as interns, which enhanced their ability to observe and contextualise participants' experiences. Before each interview, demographic information was retrieved from the ward's electronic records, and participants' functional status was assessed using the Barthel Index [25]. The concepts of resourcefulness and RT, along with the purpose of the study, were explained in detail to each patient, with assurances that their personal information would remain confidential. During the interviews, the interviewers documented the body language, tone of voice and facial expressions in real time. All interviews were transcribed promptly. In‐depth, individual interviews were considered the most appropriate method for data collection.

The first interview was conducted 1–3 days before hospital discharge in one of the two designated hospital lounges. At the end of the first session, participants' contact information and home addresses were collected with their consent to facilitate follow‐up. The second interview was conducted in the patients' homes. Before this session, the researcher reviewed the first interview transcripts to reacquaint themselves with each participant's condition, caregiver characteristics and family background. During the interviews, participants were encouraged to elaborate on previously emerging themes, supporting an iterative process rather than merely repeating data in a cross‐sectional manner [26]. Each interview lasted between 30 and 60 min, following which participants received either an electronic blood pressure monitor or a thermos flask as a token of appreciation. Data collection was discontinued once no new themes could be identified [27].

### 2.4 Data Analysis

Thematic analysis was conducted following Saldana's prescribed guidelines for longitudinal qualitative studies [28, 29] (Appendix 2). Data from each interview point were first analysed independently to ensure thematic saturation. Subsequently, data from both time points were analysed together using co‐temporal analysis, which focused on identifying the changes between the two interviews.

All interviews were transcribed within 24 h by two interviewers, who replayed the recordings sentence by sentence and cross‐checked the transcripts against their field notes. Before coding, each participant was assigned a sequential identifier to ensure anonymity. All authors engaged in reflective journaling to document their assumptions, preconceptions and personal values. Coding was conducted independently and concurrently by two coders, and all transcripts were imported into NVivo 12 (QSR International, Doncaster, Australia) for manual, tree‐structured coding. Guided by resourcefulness theory, the coding framework uses its personal and social resourcefulness structure as a priori codes to ensure comprehensive data mapping [30]. The combined dataset from both interviews yielded 749 initial codes, which were refined and consolidated into 65 distinct codes through iterative processes of definition, comparison and renaming. After coding, the two coders compared the results, and discrepancies were resolved through team discussions to integrate diverse perspectives. Themes and sub‐themes were subsequently developed and peer‐reviewed by experienced qualitative researchers who were familiar with resourcefulness theory but independent of the study team. Finally, participants reviewed the findings and provided feedback. The content was then translated into English and verified for accuracy by a bilingual researcher.

### 2.5 Rigour

All interviews were conducted by L.Y.L. and W.Y.M. (female postgraduate researchers), both of whom had received systematic training in qualitative research. Data reliability was enhanced through prolonged engagement with participants, continuous observation and documentation of their needs, and triangulation of interview data with observational field notes [31]. The study adhered to the Consolidated COREQ checklist (Appendix 3) and provided detailed descriptions of the research process and findings. This transparency enhances the credibility, transferability and applicability of results across diverse contexts [32].

## 3 Results

The first interview included 28 young and middle‐aged patients with stroke and 18 caregivers. In the second interview round, two patients and their caregivers were unable to participate in face‐to‐face interviews due to living in distant cities. Additionally, four patients and three caregivers declined to participate, and one patient could not be reached. Ultimately, 21 patients and 13 caregivers voluntarily participated, supplemented by 4 additional patients and 1 caregiver recruited after information saturation. All participants completed two interviews: the first conducted 1–3 days before discharge (T1) and the second 7–9 weeks after discharge (T2), yielding a total of 68 interviews (34 at each time point). Each interview lasted an average of 39 min (range: 30–55 min). Table 3 presents the characteristics of the stroke survivors, whose ages ranged from 35 to 64 years. More than half of the patients were male (57%, *n* = 12), 90% (*n* = 19) had ischaemic stroke and 38% (*n* = 8) did not have caregivers. The demographic information of the caregivers (*n* = 13) is summarised in Table 4.

**Table 3:** Demographic information of stroke survivors (*N* = 21).

Number	Gender	Age	Stroke type	Education	Spouse	Barthel Index		T1 and T2 Total interview duration (min)
						T1	T2	
N1	F	57	Ischaemic	Middle school	Yes	65	80	69
N2	M	53	Ischaemic	High school	Yes	60	75	75
N3	M	56	Haemorrhage	Middle school	Yes	55	65	92
N4	M	63	Ischaemic	Middle school	Yes	65	85	67
N5*	F	59	Ischaemic	Primary school	Yes	70	75	80
N6*	F	46	Ischaemic	Middle school	Yes	70	85	83
N7	M	58	Ischaemic	High school	No	65	75	70
N8	F	59	Ischaemic	Middle school	Yes	70	85	78
N9*	M	61	Ischaemic	Middle school	Yes	75	90	89
N10	F	59	Ischaemic	Primary school	No	65	75	64
N11*	M	52	Ischaemic	Middle school	Yes	75	85	66
N12	M	54	Haemorrhage	Middle school	Yes	60	75	71
N13*	M	35	Ischaemic	Professional education	Yes	75	100	90
N14*	M	51	Ischaemic	Middle school	Yes	80	95	72
N15	M	60	Ischaemic	Primary school	Yes	65	85	89
N16	F	64	Ischaemic	Middle school	Yes	65	75	70
N17*	F	58	Ischaemic	Undergraduate	Yes	70	85	92
N18	M	47	Ischaemic	High school	Yes	65	85	69
N19	F	55	Ischaemic	Middle school	Yes	75	95	105
N20	M	61	Ischaemic	Primary school	Yes	70	80	79
N21*	F	39	Ischaemic	Undergraduate	Yes	80	100	86

*Note:* Each interview averaged 39 min, yielding a total mean of 78 min for the two sessions (T1 + T2).

* Patients without caregivers.

**Table 4:** Demographic information of caregivers (*N* = 13).

Number	Gender	Age	Education	Kinship	Health status	T1 and T2 total interview duration (min)
C1	M	58	High school	Husband	Good	72
C2	F	51	Middle school	Wife	Diabetes	78
C3	F	56	Middle school	Wife	Good	69
C4	F	60	Primary school	Wife	Hypertension	60
C5	F	35	Undergraduate	Daughter	Good	92
C6	M	57	High school	Husband	Diabetes	76
C7	F	34	Professional education	Daughter	Good	85
C8	F	50	Middle school	Wife	Good	79
C9	F	57	Primary school	Wife	Good	95
C10	M	64	Middle school	Husband	Hypertension	70
C11	F	45	Professional education	Wife	Hypertension	76
C12	M	55	High school	Husband	Good	70
C13	M	39	Undergraduate	Son	Good	85

*Note:* Each interview averaged 39 min, yielding a total mean of 78 min for the two sessions (T1 + T2).

Table 5 summarises the four major themes and eight sub‐themes. The need for RT during the transition period evolved across two distinct stages, reflecting patients' psychological states and circumstances at different points in rehabilitation.

**Table 5:** Major themes and sub‐themes.

Phased	Major themes	Sub‐themes
Stage I The initial vulnerable window after discharge	Crisis awareness (Personal Resourcefulness)	Limited coping capacity
		Concerns regarding stroke recovery
	Low participation in rehabilitation training (Social Resourcefulness)	Limited initiative towards rehabilitation
		Insufficient awareness of available social resources
Stage II The open period for rebuilding the rhythm of life	Repositioning needs (Personal Resourcefulness)	Adjustment and reconstruction of self‐perception
		Along with a desire for self‐actualisation
	Desire for social support (Social Resourcefulness)	Assistance with behavioural change
		Pursuit of community acceptance

Stage I: The initial vulnerable window after discharge—emphasising personal resourcefulness.

Stage II: The open period for rebuilding the rhythm of life—transition towards social resourcefulness.

Each stage encompassed two major themes and four sub‐themes.

### 3.1 Crisis Awareness

This theme captures how, through brief but structured learning during hospitalisation, patients became aware of their limited knowledge regarding disease management. They recognised the disadvantaged and potentially risky situations they faced, along with an urgent desire to enhance their resourcefulness in coping with the progression of their condition.

#### 3.1.1 Limited Coping Capacity

Young and middle‐aged patients with stroke generally demonstrate limited awareness of stroke, which translates to insufficient coping skills. This highlights the need for training in remedial self‐control and problem‐solving strategies.

I have always been in good health, so I do not understand why I suddenly fell ill. I had no choice but to accept this fact. However, I have not had the time to learn more about it before being discharged from the hospital, so I do not have the opportunity to learn about it. (Shakes head)

N4, N9, T1

Is this disease related to blood pressure? I have been taking blood pressure medication … but I do not pay much attention to my diet. Does this have a significant impact on the disease?

N1, T1

When preparing for discharge, the doctor and nurse taught him rehabilitation exercises and healthy eating habits, but after returning home, he forgot most of it within a week and could not stick with it.

C2, T2

I know that smoking and drinking are not good for your health, but you have to do it for work and socializing, and besides, I'm young and recover faster.

N13, N18, T1

#### 3.1.2 Concerns Regarding Stroke Recovery

In terms of perceived self‐efficacy, individuals who had experienced a stroke quickly transitioned from initial shock and fear following disease onset to anxiety about recurrence and uncertainty about the future. Their experiences underscored the importance of strengthening personal resourcefulness and building confidence in the recovery process.

I feel worried and scared. When I feel unwell in the hospital, you can come to me immediately, but what will I do after I am discharged? Can I keep your phone number? (Sighs)

N16, T1

I have been in a constant state of anxiety since I became sick. My doctor told me to go home and recover, but I still could not feel my legs. I am terrified that it will be like this for the rest of my life. (Frown)

N5, T1

He has been feeling very down lately and does not like to express himself. He wanted to recover as soon as possible, but the doctor said it would take time. I am doing my best to comfort him.

C4, T1

Those first few days out of the hospital were the most helpless I have ever felt, and my quality of life took a big hit. But recovery at home is happening, just slowly—that is what I tell myself for comfort.

N2, T2

### 3.2 Low Participation in Rehabilitation Training

This suggests that stroke survivors generally demonstrate low social resourcefulness in the early stages of rehabilitation. When seeking social support, they often exhibit limited initiative, insufficient awareness, and low willingness to engage, partly due to the lack of structured guidance aimed at strengthening their social resourcefulness.

#### 3.2.1 Limited Initiative Towards Rehabilitation

Due to passive care during hospitalisation, limited self‐care awareness and reduced social interaction, young and middle‐aged patients with stroke often neglect their own initiative in rehabilitation. This, in turn, restricts their participation in RT.

Community rehabilitation center? The nurse did not mention it, so I definitely did not know about it…. And even if there was one, do I have to apply for it myself? That is too much trouble.

N7, N20, T1

My friends sent me a message asking me to join them for dinner, but I pretended not to see it…. Now, I am limping and drooling when I talk, and I do not want them to see me like this.

N13, T1

I took care of her in the hospital, but after she was discharged, I had to return to work and no longer had time to accompany her. In the first week, she locked herself in her room and said she could not do anything. (Sighs)

C5, T2

During clinical rounds, the interviewer observed that female patients were more likely to rely on their spouses or children. For example, when a nurse demonstrated post‐discharge rehabilitation exercises to participant N16, she remarked that her daughter had a good memory and would teach her after learning the exercises.

#### 3.2.2 Insufficient Awareness of Available Social Resources

Because of their limited understanding of available rehabilitation resources and social services, stroke survivors often encounter barriers to accessing social support. This lack of awareness substantially constrains their engagement in RT.

I did not know that the community offered free wheelchair rentals. I always thought I had to buy one myself. I discovered this only after asking the nurses. Otherwise, I would have taken it with me when I was discharged.

N8, T2

I have heard of community rehabilitation centers, but I do not know their exact locations or how to contact them. I do not want to try them because I am afraid it will be too troublesome. I can perform rehabilitation at home after discharge, so I think it is the same wherever I go. (shrugs)

N11, T1

I rarely participate in community activities because I worry that I may not be eligible for these rehabilitation activities or that I will have to pay additional fees.

N9, N21, T1

### 3.3 Repositioning Needs

This theme reflects the psychological shift among young and middle‐aged patients with stroke, who gradually move from fear of illness towards a stronger need for self‐identity and self‐worth. Recognising and supporting these needs through RT is essential for fostering sustained engagement in systematic rehabilitation and self‐reconstruction.

#### 3.3.1 Adjustment and Reconstruction of Self‐Perception

Stroke survivors often demonstrate a rapid acceptance of the physical and functional changes caused by their illness. At this stage, they seek to reassess their physical condition and functional limitations to establish a realistic and stable self‐image.

Right now, I just want someone to tell me clearly—how much of my left hand can recover? What tasks will I be able to do in the future, and which ones will I have to give up? I need a physical condition manual so I can adjust my expectations to the right level, instead of constantly oscillating between what if it fully heals and it is completely ruined every day.

N12, T2

Looking in the mirror at the paraplegic half of my body every day, I must admit that I am not the same as I used to be. Now, I have to rethink what state I am in and what level I can reach in the future. (Laughing bitterly.)

N3, T2

During follow‐up visits, the researchers observed that participant N18 gradually developed a consistent habit of performing rehabilitation exercises. He created a personalised rehabilitation plan, documented his progress, and expressed a desire to overcome previous limitations and move beyond the shadow of his illness.

#### 3.3.2 Along With a Desire for Self‐Actualisation

Despite acknowledging their physical and functional limitations, stroke survivors expressed a strong desire to redefine their personal values and life goals. This theme highlights the role of RT in fostering psychological resilience and addressing the low self‐esteem often associated with chronic illness.

I have been staying with relatives in the city for a while, and they take good care of me. But I always feel like I am causing trouble for others after getting sick. Sometimes I feel useless, as if everyone around me must dislike me. (Lower head, presses lips together.)

N8, T2

She has a quick temper, but since she got sick, she often stares into space, said the husband of patient N19 softly. She wants to return to work and earn money to contribute to the family.

C12, T2

He used to be the best presenter in the company, but now he speaks slowly, his hands shake, and he looks like a deflated balloon. I sincerely hope the hospital could offer him a course called Flying with Broken Wings—teaching him how to use voice‐to‐text to create PowerPoint presentations and edit videos with one hand. As long as he can see that he still has value, he will be motivated to continue rehabilitation.

C3, T2

### 3.4 Desire for Social Support

At this stage, stroke survivors demonstrated an increased need for social support and a strong desire to reintegrate into society. They gradually transitioned from passive care recipients to collaborative managers of their own health, becoming more proactive in seeking assistance from external resources. Together, these two sub‐themes reflect the key drivers of their outward growth and re‐engagement with the broader social environment.

#### 3.4.1 Assistance With Behavioural Change

Stroke survivors reported difficulty maintaining long‐term rehabilitation behaviours through willpower alone. They emphasised the critical role of external support networks, including family members, peers and healthcare professionals, in helping to translate medical advice (e.g., smoking cessation, regular exercise and healthy eating) into sustainable daily routines. In the absence of such support, adherence to rehabilitation plans often declined within 1–2 weeks.

The doctor told me to walk 6,000 steps every day, but I feel nervous when I go out alone. If there were a walking group in my neighborhood with people like me, we could meet after dinner every day to take a stroll and check in with each other. I am sure I could stick with it.

N10, T2

There are many elderly people in parks, and it is almost impossible to find young patients like me exercising there, so I always feel out of place. It would be great if I could meet other patients in the community, share their rehabilitation experiences, and encourage each other.

N17, T2

There are rules in the hospital that prohibit smoking, but once he gets home, no one can control him. He just does not have enough willpower, so he needs someone to supervise him!

C7, C9, T2

#### 3.4.2 The Pursuit of Community Acceptance

As their physical condition stabilised, their commitment to RT increased notably. They expressed a strong desire for barrier‐free facilities, accessible rehabilitation centres, and opportunities for equal treatment and interaction with neighbours, colleagues and volunteers. These elements were viewed as essential for supporting their reintegration into the community.

Every day, I see community activities outside my window, and I feel eager to participate. Once, I tried going to the community activity center, but I did not know what activities were suitable for me, and the staff did not introduce any to me. I felt awkward and left after a while.

N6, T2

He trains very seriously, but the nearest rehabilitation room is three subway stops away from home, and the round trip exhausts him. If the community could set aside even half a room for two exercise machines and open it just twice a week, his training could improve dramatically.

C11, T2

## 4 Discussion

Guided by the resourcefulness theory framework, this longitudinal qualitative study mapped the evolving RT needs of young and middle‐aged stroke survivors during the hospital‐to‐home transition. Over the approximately 2‐month follow‐up period, the demand for RT did not increase linearly but evolved subtly along a fluid and individualised trajectory. Two actionable intervention windows emerge: an initial post‐discharge vulnerability window and a subsequent open period for re‐establishing life rhythms. Across these overlapping and flexible psychosocial stages, training needs dynamically moved from personal to social resourcefulness, yielding a testable ‘phase‐cutoff’ hypothesis to inform future intervention trials. A recent trajectory study using latent class growth modelling over a 12‐month follow‐up period found that stroke patients' resourcefulness peaked approximately 1 month after discharge, followed by marked fluctuations [33], highlighting both individual variability and dynamic change. Additionally, a systematic review reported that patients and family caregivers often experience a recurring cycle of information gaps, emotional lows and resource‐seeking behaviours between 2 weeks and 3 months post‐discharge [34]. This further demonstrates the dynamic interaction between individuals and social resources during the transition period.

Related studies have indicated that the first 2 weeks after hospital discharge represent a high‐risk period for neurological fluctuations and complications, with readmission rates reaching up to 30% and more than 80% of critical turning points occurring during this window [12, 35]. Therefore, this stage can be regarded as the initial vulnerability window. An integrated study further noted that inadequate skills and knowledge training during hospitalisation are key barriers to effective transition preparation [36]. Fragmented health education during hospitalisation often leaves patients with stroke without systematic knowledge of their condition, amplifying their sense of crisis regarding recurrence and disability. The constant emergency pressure on domestic stroke centres further limits opportunities for comprehensive patient education, weakening patients' understanding of their illness and diminishing their self‐efficacy [37]. Consequently, the need for personal RT has become particularly urgent. Nursing staff and caregivers enhance patients' confidence in recovery and prevent learned helplessness by helping them access accurate disease‐related information, develop self‐management skills, and master coping strategies for specific situations [38]. At the same time, survivors' capacity to mobilise social resources during this stage is often limited. This reflects both the inherent lag in activating social support networks and highlights the social vacuum commonly experienced in the early post‐discharge period [39]. When community resources fail to be mobilised promptly, patients' relevant needs remain unmet. Based on the self‐determination theory, they must rely entirely on themselves or close relatives to maintain their motivation for rehabilitation [40]. Therefore, stroke survivors often rely heavily on family members and demonstrate limited initiative, underscoring the need for healthcare professionals and families to jointly develop detailed discharge plans and initiate face‐to‐face or online rehabilitation guidance, such as the digital follow‐up advocated by Delvallée et al., to bridge communication gaps between clinicians and patients, mitigate excessive dependence, and promote continuity of rehabilitation services [41, 42].

Research indicates that once physiological indicators stabilise, secondary stressors, such as disease‐related stigma and depression, may emerge, significantly increasing patients' need for external support [43]. Consistent with the findings of this study, psychological reconstruction and external support during this phase became the cornerstone of successful rehabilitation. However, insufficient long‐term care resources in the community and a weak informal support system can further undermine patients' sense of self‐identity and social participation after discharge [44]. Family caregivers play a critical role by closely monitoring the patient's psychological state, facilitating the expression of inner needs, encouraging participation in social activities, and supporting the patient in achieving a sense of self‐worth [39, 45]. Amalia et al. [46] argue that the lack of sustained and balanced rehabilitation resources in the community—resulting in inequalities in long‐term care—contributes to the marginalisation and abandonment of stroke survivors after discharge. Community rehabilitation resources were particularly effective during this stage. Establishing comprehensive community rehabilitation institutions, providing rehabilitation sites close to home, facilitating peer groups, and offering online mutual assistance can help mitigate social isolation and strengthen personal resourcefulness [47].

Notably, our two rounds of observation and interviews revealed multiple factors influencing the differences in RT needs among young and middle‐aged stroke survivors. Age emerged as a key factor: younger participants, with greater cognitive flexibility and active social networks, were able to shift to a positive mindset more quickly and demonstrated stronger adaptability. Related studies suggest that younger patients generally possess greater resourcefulness, mobilise social resources more effectively, and actively engage in rehabilitation training, thereby coping better with the challenges brought about by stroke [48, 49]. Furthermore, female patients appeared to be more dependent on their families during the early stages, and they utilised this reliance as a channel for emotional expression, quickly mobilising external support by communicating and listening. In the second stage, they surpassed male patients in terms of environmental adaptability. This finding aligns with the evidence that women are more willing to discuss and share their feelings. In a study analysing sex differences in secondary stroke prevention, researchers found that female patients were more inclined to express themselves, accept social support, and leverage a rich lifestyle to take their minds off the illness; they also reported a stronger sense of life satisfaction [50]. Additionally, the Barthel Index serves as a key determinant of a patient's need for RT. Higher levels of self‐care ability are associated with a more open mindset, greater social reintegration and stronger persistence in training, creating a positive feedback loop [51].

To the best of our knowledge, this is the first longitudinal qualitative study to describe how the RT needs of young and middle‐aged stroke survivors evolve and shift between personal and social domains during the transition from hospital to home. These findings will contribute to the precise implementation of rehabilitation interventions during the transitional phase of stroke recovery. Future research should combine wearable devices with multipoint tracking to evaluate the long‐term effects of interventions on recurrence rates and quality of life. A mixed‐methods design is recommended, incorporating randomised controlled trials informed by qualitative insights, to quantify the added value of dynamic RT compared to conventional health education. By integrating RT into the standard stroke transition pathway, we hope to help young and middle‐aged stroke survivors rebuild themselves, reintegrate into society, and return to work as efficiently as possible.

The methodological strength of this study lies in its combination of longitudinal interviews and participatory observations, such as in‐depth ward rounds and family scene interviews conducted by interns, anchoring RT needs in the real‐life context of ward interactions and family life. This innovative longitudinal design revealed the dynamic trajectory of resourcefulness needs among young and middle‐aged patients with stroke, overcoming the limitations of previous cross‐sectional studies and offering a new perspective for designing dynamic, phased RT interventions.

This study had some limitations. First, participants were recruited from a single tertiary hospital. Although the participants were demographically diverse, the use of a single recruitment site may limit the generalisability of the findings. Future studies should involve participants from multiple centres across different regions to validate these findings. Second, caregiver interviews primarily included spouses or children, excluding professional caregivers or social workers who may have overlooked the role of formal support systems. Finally, the health status of caregivers themselves may have influenced their assessment of patients' needs.

## 5 Conclusion

The complex challenges of hospital‐to‐home transition increase the need for RT in young stroke survivors. This longitudinal study revealed that personal and social resourcefulness needs do not remain static but follow a dynamic trajectory, shifting from personal to social resourcefulness over time. Healthcare providers should prioritise enhancing patients' cognitive self‐management skills during the early stages of recovery and gradually integrating social support resources as patients transition to home care. Intervention plans should be dynamically tailored according to age, sex and rehabilitation level to optimise care quality and improve rehabilitation outcomes for young and middle‐aged stroke survivors during the hospital‐to‐home transition.

**Table:** List of abbreviations.

Abbreviation	Full name	First appearance chapter
RT	Resourcefulness training	Abstract/Introduce
T1	Time 1	Methods
T2	Time 2	Methods
COREQ	Consolidated Criteria for Reporting Qualitative Research	Methods

## Author Contributions

**Juan Sun:** funding acquisition, data curation, formal analysis, project administration, methodology, resources, supervision, writing – review and editing. **Yali Li:** conceptualisation, data curation, formal analysis, investigation, visualisation, writing – original draft preparation, validation. **Yongmei Wang:** data curation, investigation, validation. **Yilin Du:** formal analysis, visualisation. **Hui Shen:** methodology, software, validation. **Sijia Zhang:** conceptualisation, software, supervision. **Hongru Wang:** funding acquisition, project administration, resources, supervision. **Huimin Zhang:** conceptualisation, data curation, formal analysis, methodology, resources, supervision, writing – review and editing.

## Ethics Statement

Before the start of the study, the researchers completed qualitative research training and received approval from the Ethics Committee of Xinxiang Medical College (XYLL‐20240352). The study adhered to the principles outlined in the Declaration of Helsinki. The researchers introduced themselves to the specialised nurses in each unit, clarified the purpose of the survey, arranged interviews with consenting participants, obtained signed informed consent forms, and recorded the interviews. Participants adhered to the principle of voluntarism throughout the study and were allowed to withdraw at any time.

## Consent

In this study, patients were evaluated before the investigation, and those who agreed to participate signed an informed consent form before the study commenced.

## Conflicts of Interest

The authors declare no conflicts of interest.

## Supporting information

Supporting materials for review and publication.

## Data Availability

As per ethical requirements, participant data will remain confidential. Anonymised data, including qualitative analysis, can be shared with an academic research team upon reasonable request to the corresponding author.
