# Supplementary material for: A Longitudinal Qualitative Study of Resourcefulness Training Needs During the Hospital‐to‐Home Transition After Stroke in Young and Middle‐Aged Adults
Source: Health Expect. 2025 Oct 29;28(6):e70462. doi: 10.1111/hex.70462 (PMC12569526; doi:10.1111/hex.70462)
Supplement: Supplementary file 1 — Supporting materials for review and publication. [file HEX-28-e70462-s001.docx]

**Appendix 1：Ethical Certification**

**
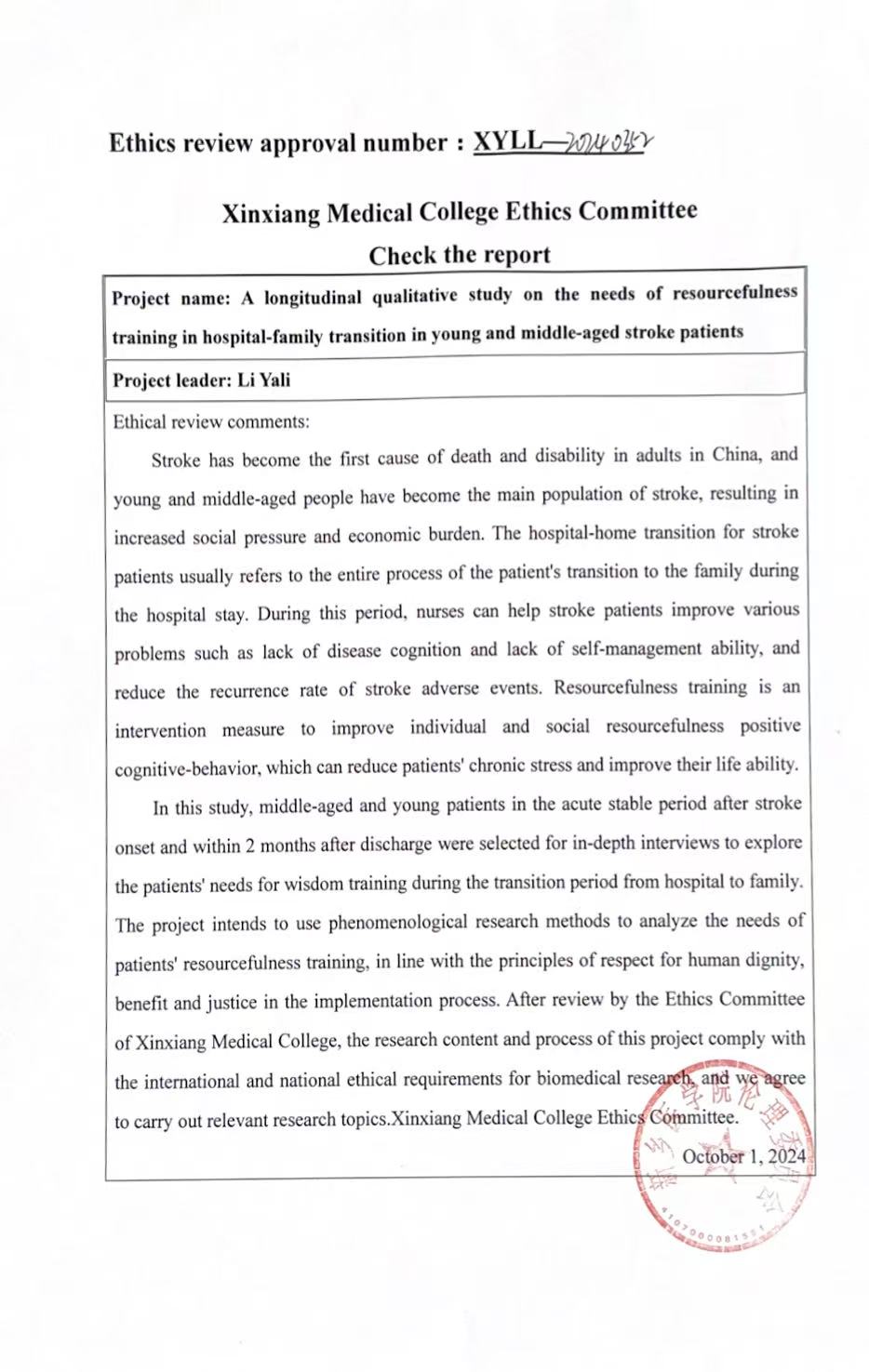
**

**Appendix 2：Saldana's prescribed guidelines for longitudinal qualitative studies**

| **Framing Questions** | What is different from one pond or pool of data through the next? |
| --- | --- |
|  | When do changes occur through time? |
|  | What contextual and intervening conditions appear to influence and affect participant changes through time? |
|  | What are the dynamics of participant changes through time? |
|  | What preliminary assertions about participant changes can be made as data analysis progresses? |
| **Descriptive Questions** | What increases or emerges through time? |
|  | What is cumulative through time? |
|  | What kinds of surges or epiphanies occur through time? |
|  | What decreases or ceases through time? |
|  | What remains constant or consistent through time? |
|  | What is idiosyncratic through time? |
|  | What is missing through time? |
| **Analytic and Interpretive Questions** | Which changes interrelate through time? |
|  | Which changes through time oppose or harmonize with natural human development or constructed social processes? |
|  | What are participant or conceptual rhythms through time? |
|  | What is the through-line of the study? |

**Appendix 3：COREQ** **(Consolidated** **criteria** **for** **Reporting** **Qualitative** **research)** **Checklist**

A checklist of items that should be included in reports of qualitative research. You must report the page number in your manuscript where you consider each of the items listed in this checklist. If you have not included this information, either revise your manuscript accordingly before submitting or note N/A.

| **Topic** | **Item** **No.** | **Guide** **Questions/Description** | **Reported** **on**  **Page** **No.** |
| --- | --- | --- | --- |
| **Domain** **1:** **Research** **team** **and** **reﬂexivity** | | | |
| *Personal* *characteristics* | | | |
| Interviewer/facilitator | 1 | Which author/s conducted the interview or focus group? | 8 |
| Credentials | 2 | What were the researcher’s credentials? E.g., PhD, MD | 7 |
| Occupation | 3 | What was their occupation at the time of the study? | 8 |
| Gender | 4 | Was the researcher male or female? | 10 |
| Experience and training | 5 | What experience or training did the researcher have? | 10 |
| *Relationship* *with* *participants* | | | |
| Relationship established | 6 | Was a relationship established prior to study commencement? | 6 |
| Participant knowledge of the interviewer | 7 | What did the participants know about the researcher? e.g. personal goals, reasons for doing the research | 8 |
| Interviewer characteristics | 8 | What characteristics were reported about the inter viewer/facilitator? e.g. Bias, assumptions, reasons and interests in the research topic | 8 |
| **Domain** **2:** **Study** **design** | | | |
| *Theoretical* *framework* | | | |
| Methodological orientation and Theory | 9 | What methodological orientation was stated to underpin the study? e.g. grounded theory, discourse analysis, ethnography, phenomenology,  content analysis | 6 |
| *Participant* *selection* | | | |
| Sampling | 10 | How were participants selected? e.g. purposive, convenience, consecutive, snowball | 7 |
| Method of approach | 11 | How were participants approached? e.g. face-to-face, telephone, mail, email | 8 |
| Sample size | 12 | How many participants were in the study? | 10 |
| Non-participation | 13 | How many people refused to participate or dropped out? Reasons? | 10 |
| *Setting* | | | |
| Setting of data collection | 14 | Where was the data collected? e.g. home, clinic, workplace | 8,9 |
| Presence of non- participants | 15 | Was anyone else present besides the participants and researchers? | 8,9 |
| Description of sample | 16 | What are the important characteristics of the sample? e.g. demographic data, date | 11,12 |
| *Data* *collection* | | | |
| Interview guide | 17 | Were questions, prompts, guides provided by the authors? Was it pilot tested? | 7,8 |
| Repeat interviews | 18 | Were repeat inter views carried out? If yes, how many? | 7 |
| Audio/visual recording | 19 | Did the research use audio or visual recording to collect the data? | 8 |
| Field notes | 20 | Were ﬁeld notes made during and/or after the inter view or focus group? | 8 |
| Duration | 21 | What was the duration of the interviews or focus group? | 9 |
| Data saturation | 22 | Was data saturation discussed? | 9 |
| Transcripts returned | 23 | Were transcripts returned to participants for comment and/or correction? | 10 |

| **Topic** | **Item** **No.** | **Guide** **Questions/Description** | **Reported** **on**  **Page** **No.** |
| --- | --- | --- | --- |
| **Domain** **3:** **analysis** **and** **ﬁndings** | | | |
| *Data* *analysis* | | | |
| Number of data coders | 24 | How many data coders coded the data? | 10 |
| Description of the coding tree | 25 | Did authors provide a description of the coding tree? | 9,10 |
| Derivation of themes | 26 | Were themes identiﬁed in advance or derived from the data? | 9 |
| Software | 27 | What software, if applicable, was used to manage the data? | 9 |
| Participant checking | 28 | Did participants provide feedback on the ﬁndings? | 10 |
| *Reporting* | | | |
| Quotations presented | 29 | Were participant quotations presented to illustrate the themes/ﬁndings? Was each quotation identiﬁed? e.g. participant number | 13-18 |
| Data and ﬁndings consistent | 30 | Was there consistency between the data presented and the ﬁndings? | 13-18 |
| Clarity of major themes | 31 | Were major themes clearly presented in the ﬁndings? | 13-18 |
| Clarity of minor themes | 32 | Is there a description of diverse cases or discussion of minor themes? | 21-22 |

Developed from: Tong A, Sainsbury P, Craig J. Consolidated criteria for reporting qualitative research (COREQ): a 32-item checklist for interviews and focus groups. *International* *Journal* *for* *Quality* *in* *Health* *Care*. 2007. Volume 19, Number 6: pp. 349 – 357

**Once** **you** **have** **completed** **this** **checklist,** **please** **save** **a** **copy** **and** **upload** **it** **as** **part** **of** **your** **submission.** **DO** **NOT** **include** **this** **checklist** **as** **part** **of** **the** **main** **manuscript** **document.** **It** **must** **be** **uploaded** **as** **a** **separate** **file.**
